# Treatment of hypothalamic obesity in people with hypothalamic injury: new drugs are on the horizon

**DOI:** 10.3389/fendo.2023.1256514

**Published:** 2023-09-13

**Authors:** Christian L. Roth, Anna Zenno

**Affiliations:** ^1^ Seattle Children’s Research Institute, Department of Pediatrics, School of Medicine, University of Washington, Seattle, WA, United States; ^2^ Division of Endocrinology, Department of Pediatrics, University of Washington, Seattle, WA, United States

**Keywords:** obesity, brain tumor, hypothalamic injury, energy homeostasis, drug intervention

## Abstract

Hypothalamic obesity (HO) is a complex and rare disorder affecting multiple regulatory pathways of energy intake and expenditure in the brain as well as the regulation of the autonomic nervous system and peripheral hormonal signaling. It can be related to monogenic obesity syndromes which often affect the central leptin-melanocortin pathways or due to injury of the hypothalamus from pituitary and hypothalamic tumors, such as craniopharyngioma, surgery, trauma, or radiation to the hypothalamus. Traditional treatments of obesity, such as lifestyle intervention and specific diets, are still a therapeutic cornerstone, but often fail to result in meaningful and sustained reduction of body mass index. This review will give an update on pharmacotherapies of HO related to hypothalamic injury. Recent obesity drug developments are promising for successful obesity intervention outcomes.

## 1 Introduction

Hypothalamic obesity (HO) is one of the most recalcitrant examples of excessive weight gain and most commonly caused by hypothalamic lesions and tumors such as craniopharyngioma (CP), an embryological tumor located in the hypothalamic and/or pituitary region, and its treatment by surgery and irradiation (1–4). Additional etiologies include other suprasellar brain tumors, trauma, inflammation, and some genetic syndromes (2, 4, 5). CP frequently causes not only hypopituitarism, but also damages the medial hypothalamic nuclei. After surgery, hyperphagia and severe obesity, a major risk factor for craniopharyngioma-related morbidity and mortality, occurs in 50% of survivors even with optimal endocrine management of hypopituitarism (6). Mechanisms leading to the profoundly disturbed energy homeostasis are complex and not well elucidated. Early and effective management of obesity is vital for this population (7), which is more resistant to treatment than alimentary obesity (8–16). There are currently no approved drugs for treatments of HO (17), but drug interventions that tackle both reduction of hyperphagia and stimulation of thermogenesis are promising.

## 2 Hypothalamic obesity related to hypothalamic and pituitary tumors

Patients with CP frequently develop obesity following tumor therapy with surgery and/or radiation, and have more features of metabolic syndrome compared to matched controls (18, 19). Furthermore, people with CP have a 3-19-fold higher cardiovascular mortality (20, 21), as well as increased rates of cerebral infarction and type 2 diabetes mellitus compared to the general population (22). In affected patients, both quality of life and survival are substantially decreased due to these metabolic consequences (6, 23). For example, the estimated prevalence of nonalcoholic fatty liver disease in survivors of childhood craniopharyngioma is 47% (24). HO can also occur due to other suprasellar tumors, radiation, trauma, or surgical insult to the hypothalamus. The excessive weight gain following hypothalamic injury occurs irrespective of pituitary deficiency secondary to damage to the hypothalamic-pituitary axis and optimal replacement of hormones. Many, though not all, individuals with HO also experience hyperphagia (25, 26). Recognized risk factors for severe obesity include large hypothalamic tumors or aggressive resections affecting several medial and posterior hypothalamic nuclei reaching the floor of the third ventricle, the area beyond mammillary bodies, and several satiety signaling pathways (10, 27–29). Affected medial hypothalamic nuclei frequently are arcuate (ARC), ventromedial (VMN) and dorsomedial (DMN) nuclei (30). Structural damage of these nuclei often leads to hyperphagia, rapid post-operative weight gain, central insulin and leptin resistance, decreased sympathetic activity, low metabolic rate, and increased energy storage in adipose tissue (11, 30). In particular, VMN damage can lead to disinhibition of vagal tone, resulting in excess stimulation of pancreatic β-cells, hyperinsulinemia, and obesity. Several previous studies showed that the secretion of satiety regulating peptides, such as ghrelin and peptide YY (PYY), may also be altered in CP patients (31, 32).

## 3 The hypothalamic obesity phenotype

Clinical features of the full HO syndrome include excessive weight gain leading to morbid obesity with uncontrolled appetite, potentially caused by central leptin resistance and deficient downstream pathways, fatigue, decreased sympathetic activity, low energy expenditure, temperature dysregulation, and increased energy storage in adipose tissue (1–4, 13, 25, 26, 33–37). Similar clinical features are also observed in patients suffering from HO syndrome due to a genetic abnormality (i.e. melanocortin-4 receptor defect, leptin or proopiomelanocortin (POMC) deficiency, Bardet-Biedl Syndrome).

## 4 Disturbed energy balance in hypothalamic obesity

While some patients report hyperphagia, other patients have lower energy intake compared to relevant controls (25, 26). This reduction in energy intake is thought to be offset by greater relative decrease in basal metabolic rate and physical activity that leads to excessive weight gain in HO (26). Alpha-melanocyte stimulating hormone (α-MSH) is one of the key weight-regulating neuropeptides after binding to melanocortin receptor subtypes 3 and 4 in the brain; it is mainly produced in the pituitary and hypothalamus from the cleavage of POMC (38, 39). Individuals with hypothalamic lesions related to craniopharyngioma appear to have significantly decreased serum levels of α-MSH (40, 41), suggesting a melanocortin pathway deficiency. Compared to controls, individuals with HO have significantly higher baseline and post-meal circulating insulin concentrations, potentially indicating insulin hypersecretion through vagus nerve hyperstimulation (11, 31, 32). In HO, biochemically, the degree of hyperleptinemia and hyperinsulinemia are unexpectedly high for the degree of obesity, and catecholamine levels are low, suggestive of decreased sympathetic tone (26, 30, 32, 42–44). Chronic hyperleptinemia, decreased sympathetic tone and deficiency of downstream outputs of leptin signaling are key features contributing to the pathogenesis of HO. Furthermore, disturbed cicadian rhythm and narcolepsy could contribute to decreased energy expenditure (45).

## 5 Earlier obesity drug developments

**Figure 1:**
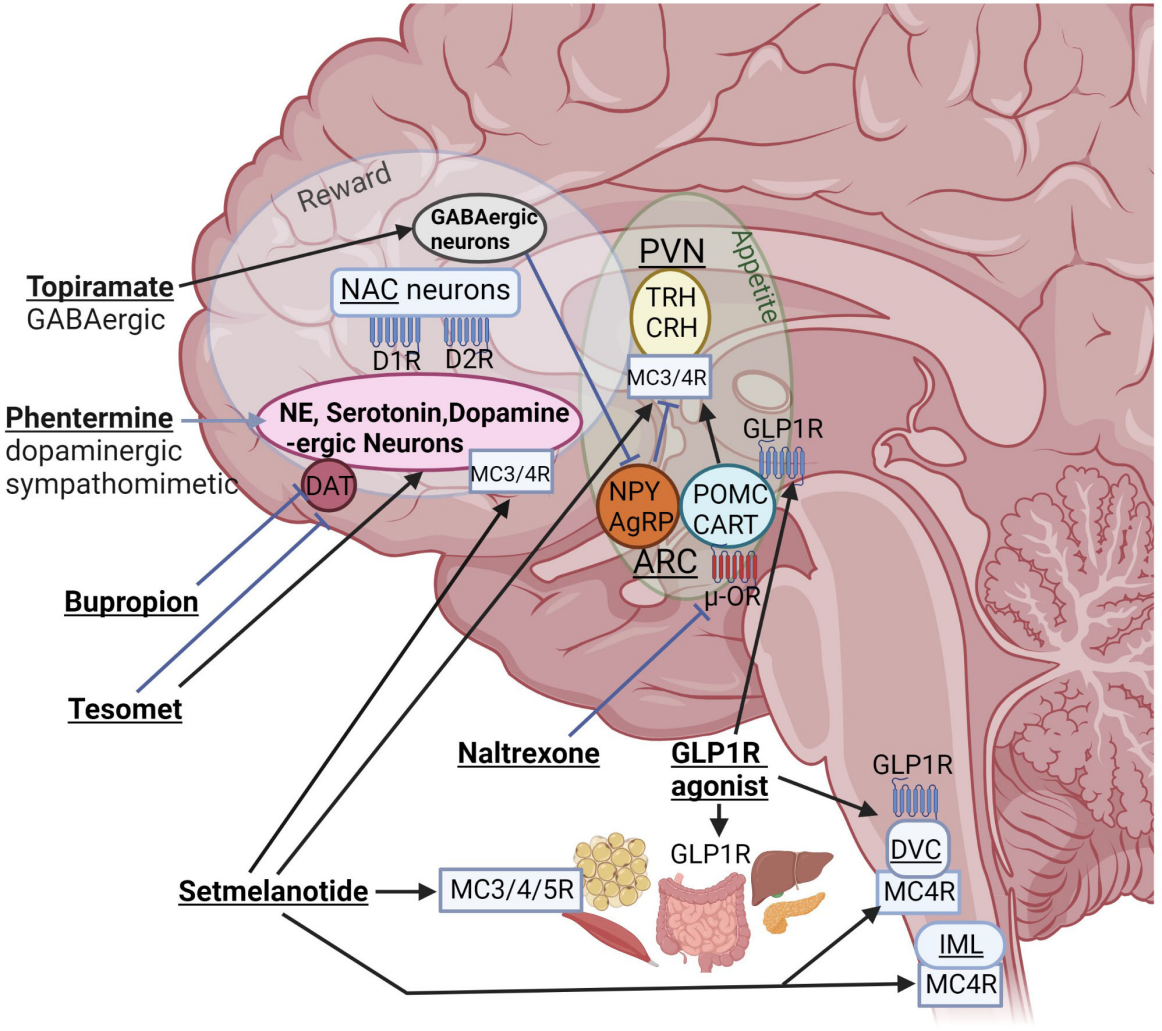
Interactions of different anti-obesity agents with orexigenic and anorexigenic pathways of the homeostatic “appetite” center and the “reward” system. Even if mediobasal hypothalamic structures such as the arcuate nucleus (ARC) and para-ventricular nucleus (PVN) are damaged, these drugs can interact with peripheral or brain receptors outside of hypothalamic structures. NPY, neuropeptide Y; AgRP, agouti-related peptide; POMC, proopiomelanocortin; CART, cocaine and amphetamine regulated transcript; NAC, nucleus accumbens; DR, dopamine receptor; DAT, dopamine active transporter; DVC, dorsal vagal complex of brainstem; IML, intermediolateral nucleus along spinal cord containing MC4R–expressing preganglionic cholinergic sympathetic neurons; blue, stimulatory and red, inhibitory receptors; TRH, thyrotropin-releasing hormone; CRH, corticotropin-releasing hormone (created with BioRender.com).

In 2003, the only FDA approved weight loss medication for adolescents was orlistat, a pancreatic lipase inhibitor, but it was rarely used due to its low efficacy (46) and unfavorable gastrointestinal side effect profile (46, 47). Several central stimulants have been used off label for the treatment of HO such as methylphenidate, phentermine, dextroamphetamine, mazindol, caffeine and ephedrine (8, 48–51), but data on these drugs came from small studies with mixed results. Other medications such as sibutramine, a norepinephrine and serotonin inhibitor, lorcaserin, a serotonin 2C receptor agonist, and beloranib, a methionine aminopeptidase 2 (MetAP2) inhibitor, were discontinued due to significant adverse events of thromboembolism and other safety concerns (14). Beloranib was initially developed as a cancer drug but its use resulted in progressive weight loss in patients and rodents with HO due to hypothalamic injury (52, 53). Its mechanism for weight loss is not fully understood, but it is believed that it increases fat oxidation and lipolysis resulting in reduced fat mass. Furthermore, it leads to a reduction in caloric intake which is likely centrally mediated. Finally, the combination of bupropion, a norepinephrine-dopamine reuptake inhibitor, and naltrexone, an opioid receptor antagonist (**Figure 1**), has a black box warning of increased suicidal risk and ideation in young adults (46). There are no studies of bupropion-naltrexone in patients with HO.

### 5.1 Glucagon-like peptide-1 receptor agonists (GLP1RAs) for HO

Besides its well described effects on glucose metabolism (54), GLP-1 also functions as a satiety hormone, promoting reduced food intake and meal termination through direct action on the vagus nerve and the brain, including the hypothalamus, hindbrain, hippocampus and mesolimbic brain reward system (55). In addition, GLP1RA treatment activates catecholamine neurons in the area postrema (56). GLP1RAs can be effective weight loss agents in HO due to their effects outside of the hypothalamus. In addition, GLP1RAs may change the balance of sympathetic/parasympathetic tone (57–59), potentially counteracting the abnormalities we have seen in HO (42). GLP1Rs are widely expressed in peripheral tissues that are important for energy regulation, such as adipose tissue, pancreas, liver, and gut (**Figure 1**). In rodents, GLP1RA semaglutide modulates food preference, reduces food intake, and causes weight loss without decreasing energy expenditure (60). In humans, GLP1RAs, such as semaglutide in the setting of type 2 diabetes (T2D) treatment, reduce the risk of cardiovascular death, nonfatal myocardial infarction and nonfatal stroke (61). Liraglutide is approved in children age 10 years and older for T2D (62), and recently semaglutide was approved for the treatment of obesity in adolescents 12 years and older, with 17% decrease in BMI with semaglutide compared with placebo (63). Liraglutide causes dose-dependent and treatment duration-dependent thyroid C-cell tumors in rodent models, at much higher dose exposures than those used in humans. Therefore, even if the relevance for humans of such tumors has not been determined, GLP1RAs are contraindicated in patients with a family history of medullary thyroid carcinoma or multiple endocrine neoplasia syndrome type 2 (64). Otherwise, gastrointestinal adverse effects are frequent in particular at start of treatment, potentially leading to discontinuation of treatment (65).

Recently, our group tested weekly exenatide in a multicenter, randomized, double-blind, placebo-controlled clinical trial in 10- to 25-year-old children and young adults with HO in the context of hypothalamic injury following intracranial tumor (ECHO trial). In this trial, study participants were randomized to once weekly subcutaneous injections of exenatide 2 mg or placebo for 36 weeks, followed by an 18 week open label once weekly exenatide 2 mg extension for all participants. Modest effects were seen on BMI reduction, while there was a significant reduction of body fat measured by DXA and waist circumference in the exenatide group vs. placebo after 36 weeks (66, 67). Overall, exenatide was well tolerated with the majority of adverse events related to gastrointestinal problems such as nausea and vomiting. In this study, the degree of hypothalamic damage was assessed using a hypothalamic lesion score (HLS). Patients with more extensive hypothalamic damage with involvement of the mammillary bodies showed greater reductions in adiposity following GLP1RA treatment (68). One possible explanation for these study findings is that destruction of endogenous ligand sites of action in the hypothalamus heightens responsiveness of extra-hypothalamic sites of action to exogenous ligands such as GLP1RAs. A clinical trial with newer and more potent GLP1RAs has not yet been performed in patients with HO. Furthermore, incretin-based multiple agonists, such as dual GLP-1/glucose-dependent insulinotropic polypeptide (GIP) receptor agonist tirzepatide or triple GIP/GLP-1, and glucagon receptor agonist retatrutide, are promising for treatment of HO, as multiple redundant weight regulatory pathways are simultaneously targeted (see section 1.7), potentially preventing compensatory weight regain.

## 6 Recent drug discoveries

### 6.1 Therapeutic potential of exogenous oxytocin for “common” obesity and HO

Oxytocin (OXT) is a 9-amino acid peptide made in the hypothalamic paraventricular and supraoptic nuclei. Its effect has been tested outside its classical use during labor, and a substantial body of literature supports its favorable safety profile in a wide range of conditions, mostly neuropsychiatric (69).

The neuropeptide OXT induces appetite suppression and acts *via* brain reward centers and peripheral receptors. OXT-induced weight loss may also be partly due to increased lipolysis and energy expenditure by stimulation of the sympathetic tone and adipose tissue thermogenesis (70). The potential role of OXT as a therapy for obesity has been recently investigated (71). A recent study demonstrated that a single dose of intranasal OXT leads to decreased caloric intake at a subsequent meal in men who are lean, overweight, and with obesity (72). Moreover, in a randomized controlled trial of healthy adults with obesity (N=24), 8 weeks of intranasal OXT (24 IU at mealtimes and bedtime) led to an 8.9 kg weight loss (73).

OXT status has been investigated in individuals with history of brain tumors affecting the hypothalamus and pituitary (70). In a small pilot study, low OXT levels were associated with higher BMI in men with hypopituitarism and diabetes insipidus, however, this finding did not reach statistical significance (74). In a separate study of individuals with craniopharyngioma, the change in salivary OXT in response to meals (75) and exercise (76) appeared to be related to BMI and eating behaviors (77), thereby demonstrating a potential utility of OXT in HO. Furthermore, in a case report of a child with hypopituitarism, diabetes insipidus, and obesity following therapy for a craniopharyngioma, low dose intranasal OXT 6 IU daily combined with carbohydrate restriction, and naltrexone (**Figure 1**) led to amelioration of hyperphagia, and a sustained decrease in BMI (78). A recent randomized placebo-controlled clinical trial was conducted to determine whether 8 weeks of intranasal OXT (vs 8 weeks of placebo) promotes weight loss in children, adolescents, and young adults with HO using a cross-over design. In this small pilot study with 10 completers, there was no significant effect of intranasal OXT on body weight change. However, OXT was well tolerated, and in exploratory analyses, benefits of OXT for anxiety and impulsivity were noted (79). Preclinical research using labeled OXT administered intranasally to rhesus macaques or rabbits reached the cerebrospinal fluid and brain (80, 81). It is unclear, to which extent weight reduction in response to peripherally or intranasally administered OXT is related to peripheral or central effects. Individuals with hypothalamic injury might lack the ability to respond to intranasal OXT because key hypothalamic nuclei that impact appetite and energy balance are missing. In animal models, however, administration of OXT outside the hypothalamus, in the hindbrain or peripherally, decreases weight gain *via* decrease in food intake and activation of catecholamine neurons in nucleus tractus solitarius (NTS, see **Figure 1**) (70, 82–84).

Therefore, OXT is promising, as it has the potential for reducing energy intake but also increasing energy expenditure by increased brown adipose tissue thermogenesis which is important for avoiding compensatory adaptations to weight loss. However, there are many open questions regarding the mechanisms of action, optimized dosing, and individual factors such as genetics, which might have an impact on responses to OXT treatment.

### 6.2 The combination of oral phentermine and topiramate (Ph/T)

The combination of oral phentermine and topiramate (Ph/T) is another promising option, which is FDA-approved for people with obesity ≥12y (85, 86). However, this drug has not yet been tested in HO. Ph/T is a sympathomimetic amine combined with a GABA-ergic drug used to treat epilepsy (**Figure 1**). Children and adolescents with HO have low sympathetic tone (42, 87), thus may benefit from the stimulant-induced decrease in appetite (15, 50). Individuals with HO can also have excess daytime sleepiness (87, 88) which may contribute to impaired regulation of eating behavior and decreased physical activity (26), and may be targetable with stimulants. Reports of other types of stimulant use in individuals with HO have generally demonstrated weight loss or attenuation of weight gain (48, 87, 89). Sibutramine, a compound related to Ph, led to a decrease in BMI in children with HO (14). However, these previous reports demonstrated heterogeneity between participants, and an overall modest effect. Thus, combining Ph with T, may increase the potential maximal weight loss for treatment of HO.

### 6.3 Tesofensine for treatment of hypothalamic obesity

Tesofensine is a centrally acting triple monoamine reuptake inhibitor that tackles low sympathetic tone in patients with HO. Tesofensine results in reduction of caloric intake and weight loss by inhibiting the presynaptic reuptake of dopamine, serotonin, and noradrenaline, and inhibiting the dopamine active transporter (90) (**Figure 1**). It is combined with the β-blocker metoprolol to reduce potential adverse effects due to adrenergic stimulation. Results from rodent studies suggest that indirect α-adrenergic and D1-dopaminergic stimulation contribute to reduction of food intake and body weight (91). In a recent randomized, double-blind, placebo-controlled Phase 2 trial, Tesomet (tesofensine 0.5 mg + metoprolol 50 mg) or matching placebo was administered daily following a 2:1 randomization for 24 weeks, followed by an open-label extension of another 24 weeks for a total of 48 weeks of treatment. Twenty-one adults with HO (16 females) were randomized, and 18 patients completed 24 weeks of treatment. Tesomet treatment resulted in significant reductions in body weight, waist circumference and glucose levels compared to placebo (92), which were the main efficacy endpoints. The study results also showed that Tesomet was safe and well tolerated and drug-related adverse events were mostly mild sleep disturbances, dry mouth, and headache. There were no significant differences in heart rate or blood pressure between Tesomet and placebo groups (92). A larger clinical trial has not yet been performed testing Tesomet in HO, but a Phase 3 clinical trial is planned.

### 6.4 Melanocortin (MC) pathway in the regulation of energy homeostasis and glucoregulation

The MC pathway plays a central role in the regulation of energy homeostasis (93). The MC system consists of two distinct neurons expressed in the arcuate nucleus that express POMC and agouti-related protein (AgRP). These neurons mediate opposing effects on downstream neurons that express the MC-4 receptor (MC4R). POMC neuron activation induces synaptic release of α-melanocyte stimulating hormone (α-MSH - an MC4R agonist), while AgRP is an inverse agonist that binds to and blocks signaling by MC4R (38, 39). The MC4R is one of five human MC receptor subtypes (MC1−5R) that share class A G-protein-coupled receptors (94). The receptors are critically involved in the regulation of energy homeostasis, pigmentation, cardiovascular function, and sexual functions (94). The MC4R plays a key role in energy balance and appetite regulation and is expressed in different brain areas including the paraventricular nucleus of the hypothalamus, areas of the brain reward system, the dorsal motor nucleus of the hindbrain, the preganglionic cholinergic sympathetic neurons of the spinal cord and vagal afferent nerves (**Figure 1**) (95, 96). When activated, it reduces food intake, increases energy expenditure, and regulates gut hormone secretion such as the potent anorexigenic hormone PYY_3-36_ *via* enteroendocrine cells (97). Consequently, administration of pharmaceutical agents that agonize MC4R cause weight loss by suppressing energy intake and stimulating energy expenditure, whereas genetic or pharmacological inhibition of MC4R results in hyperphagia and obesity. While most of the effects of α-MSH are mediated by the brain, it is also released into circulation from the pituitary gland (41, 98, 99), and can act on peripheral tissues, such as the enteric nervous system (100), and brown adipose tissue (101, 102). Systemic α-MSH increases muscle thermogenesis and glucose clearance by increased muscle glucose uptake (41, 97). Moreover, further evidence suggests that the MC pathway is important in the regulation of glucose metabolism, independent of its effects on energy balance. Restoring expression of leptin receptors in POMC neurons normalizes blood glucose and ameliorates hepatic insulin resistance independent of changes in body weight (103).

Synthetic MC receptor agonists are promising for treatment of genetic and surgery-induced HO affecting the MC4R pathway (104) as there is clear evidence that central MC signaling is deficient (40, 41, 44, 105–107) in patients with hypothalamic lesions. Thus, treatment with a MC receptor agonist could present as targeted treatment supplementing a deficiency. For example, setmelanotide, a MC4R agonist is FDA approved for treatment of monogenic obesity related to POMC deficiency, leptin receptor (LEPR) deficiency (108, 109), and Bardet Biedl syndrome (110). Most recently setmelanotide has also been shown to be effective in reducing hunger and BMI in patients with HO due to hypothalamic injury (111, 112), which could mark a breakthrough for the treatment of aquired HO. Side effects included nausea and vomiting. A large international multicenter Phase 3 clinical trial over 1 year investigating the use of setmelanotide in HO is currently underway.

## 7 Summary

HO is a complex and rare disorder that is characterized by excessive weight gain and difficulty in losing weight due to disruptions in the hypothalamus with resultant dysregulation of appetite and metabolism. Its treatment involves a multidisciplinary approach including dietary modifications, physical activity, and behavioral interventions, but they often fail to result in meaningful and sustained reduction of body mass index. Thus, anti-obesity pharmacotherapy has been increasingly used to promote further weight loss. The anti-obesity drugs for HO that were featured in this review were oxytocin, oral phentermine and topiramate, exenatide, tesofensine, and setmelanotide. Apart from one phase 3 randomized controlled trial that enrolled 42 participants testing weekly exenatide (66), most studies in HO are small case reports or series with fewer than 10 individuals given the rarity of this condition. Thus, current weight loss drugs for HO are being used off label with variable results and require close monitoring. New clinical trials, however, are underway to investigate the use of promising anti-obesity pharmacotherapy for treatment of HO.

## 8 Outlook

The landscape of obesity pharmacotherapy is evolving quickly with frequent development of new drugs. Examples are dual and triple agonists that target multiple receptors simultaneously to enhance the effects of various hormones involved in appetite regulation and metabolism to promote weight loss (113). Tirzepatide, a GLP-1 and GIP receptor agonist is an example of a dual agonist that was recently FDA approved for the treatment of adults with type 2 diabetes with its clinical trials showing up to 20% weight loss in participants on the 15 mg dose (114). Retatrutide is an agonist of GIP, GLP-1, and glucagon receptors making it a “triple agonist.” Recent Phase 2 trial results showed 48 weeks of retatrutide treatment resulted in substantial reductions in body weight of -24.2% in the 12-mg group (115). For successful intervention of this treatment-resistant form of obesity, personalized treatment approaches appear to be necessary. Individualized based on pathophysiology and initial treatment responses, Iersel et al. developed algorithms which may be the right direction for the treatment of HO based on phenotypic spectrum (17). If there is no beneficial effect on weight reduction after 3 months of treatment, switching to another antiobesity drug or a combination of drugs might be indicated. Currently, MC4R agonism, i.e. supplementing melanocortin deficiency, could represent a new benchmark for the treatment of HO.

## Author contributions

CR: Conceptualization, Writing – original draft, Writing – review & editing. AZ: Writing – original draft, Writing – review & editing.
